# Bloom Dynamics of Cyanobacteria and Their Toxins: Environmental Health Impacts and Mitigation Strategies

**DOI:** 10.3389/fmicb.2015.01254

**Published:** 2015-11-17

**Authors:** Rajesh P. Rastogi, Datta Madamwar, Aran Incharoensakdi

**Affiliations:** ^1^BRD School of Biosciences, Sardar Patel UniversityAnand, India; ^2^Laboratory of Cyanobacterial Biotechnology, Department of Biochemistry, Faculty of Science, Chulalongkorn UniversityBangkok, Thailand

**Keywords:** cyanobacteria, eutrophication, cyanobacterial blooms, cyanotoxins, ecotoxicology, mitigation strategies

## Abstract

Cyanobacteria are ecologically one of the most prolific groups of phototrophic prokaryotes in both marine and freshwater habitats. Both the beneficial and detrimental aspects of cyanobacteria are of considerable significance. They are important primary producers as well as an immense source of several secondary products, including an array of toxic compounds known as cyanotoxins. Abundant growth of cyanobacteria in freshwater, estuarine, and coastal ecosystems due to increased anthropogenic eutrophication and global climate change has created serious concern toward harmful bloom formation and surface water contamination all over the world. Cyanobacterial blooms and the accumulation of several cyanotoxins in water bodies pose severe ecological consequences with high risk to aquatic organisms and global public health. The proper management for mitigating the worldwide incidence of toxic cyanobacterial blooms is crucial for maintenance and sustainable development of functional ecosystems. Here, we emphasize the emerging information on the cyanobacterial bloom dynamics, toxicology of major groups of cyanotoxins, as well as a perspective and integrative approach to their management.

## Introduction

**FIGURE 1:**
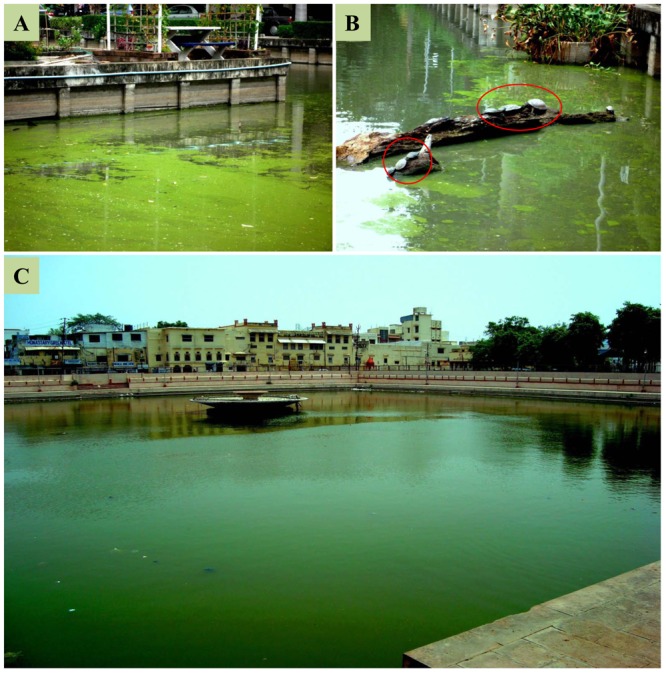
**Examples of excessive nutrient enrichment and bloom dynamics in freshwater ponds. (A)** Harmful algal blooms in a pond at Chulalongkorn University, Bangkok, Thailand, showing the life of turtles (in red circle) under the toxic blooms condition **(B)**. **(C)** Harmful algal blooms in a large pond in Varanasi, India (Photograph by R.P. Rastogi).

Cyanobacteria are considered the most primitive groups of photosynthetic prokaryotes (Bullerjahn and Post, 2014) and possibly appeared on the Earth about 3.5 billion years ago (Tomitani et al., 2006). They are ubiquitous in nature and thrive in a variety of ecological niches ranging from desert to hot springs and ice-cold water. Most cyanobacteria are an immense source of several secondary natural products with applications in the food, pharmaceuticals, cosmetics, agriculture, and energy sectors (Rastogi and Sinha, 2009). Moreover, some species of cyanobacteria grow vigorously and form a dominant microflora in terms of their biomass and productivity in specific ecosystems. Bloom formations (**Figure 1**) due to excessive growth of certain cyanobacteria followed by the production of toxic compounds have been reported in many eutrophic to hypertrophic lakes, ponds, and rivers throughout the world (Rastogi et al., 2014). A range of toxic secondary compounds, called cyanotoxins, have been reported from cyanobacteria inhabiting freshwater and marine ecosystems. These toxic compounds are highly detrimental for survival of several aquatic organisms, wild and/or domestic animals, and humans. Aquatic organisms, including plants and animals, and phyto/zoo-planktons inhabiting under toxic bloom rich ecosystems, are directly exposed to the harmful effects of different cyanotoxins. The intoxication occurring in wild and/or domestic animals and humans is either due to direct ingestion of cells of toxin producing cyanobacteria or the consumption of drinking water contaminated with cyanotoxins (Rastogi et al., 2014). The toxicity of different cyanotoxins is directly proportional to the growth of cyanobacteria and the extent of their toxin production. It has been shown that the growth of different cyanobacteria and their toxin biosynthesis is greatly influenced by different abiotic factors such as light intensity, temperature, short wavelength radiations, pH, and nutrients (Neilan et al., 2013; Häder et al., 2014; Rastogi et al., 2014). Global warming and temperature gradients can significantly change species composition and favor blooms of toxic phytoplanktons (El-Shehawy et al., 2012; Häder and Gao, 2015).

It has been assumed that cyanotoxins play an important role in chemical defense mechanisms giving survival advantages to the cyanobacteria over other microbes or deterring predation by higher trophic levels (Vepritskii et al., 1991; Jang et al., 2007; Berry et al., 2008). Cyanotoxins may also take part in chemical signaling. Overall, information regarding the specific role(s) of cyanotoxins in the life of individual cyanobacteria or their ecological and biotechnological operations is still very limited and needs extensive research. In the present review, we summarize the recent advances on bloom dynamics, cyanotoxin production, and mitigation strategies as well as their consequences on environmental health perspectives.

## Eutrophication, Global Climate Change, and Cyanobacterial Bloom Dynamics

**FIGURE 2:**
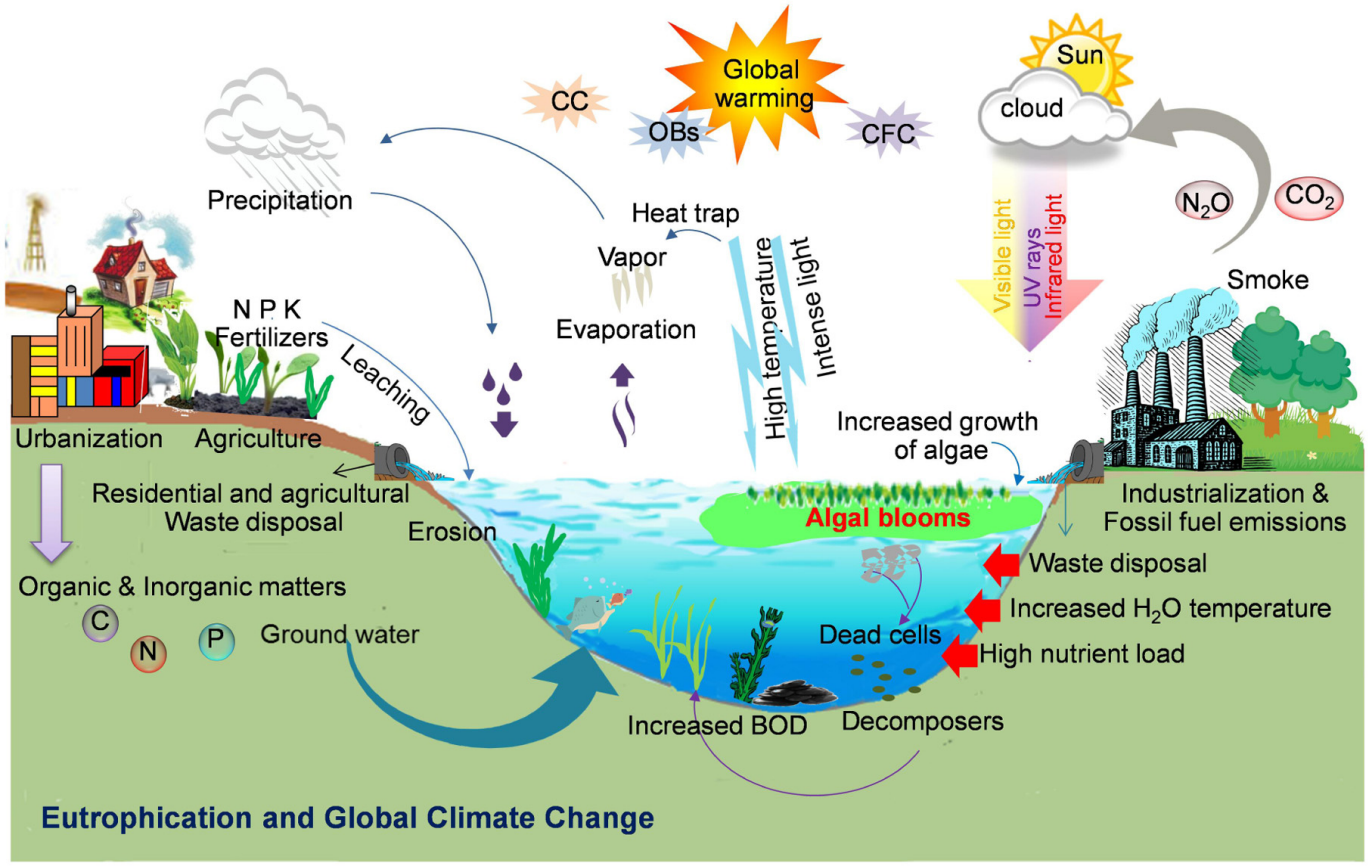
**Formation of cyanobacterial blooms: Schematic illustration showing the key factors such as anthropogenic eutrophication, global climate change such as increased temperature and light or global warming due to an increase in ozone depleting substances (e.g., CO_2_, N_2_O, etc.), and other biotic and abiotic factors responsible for the worldwide bloom incidence (Illustration by R. P. Rastogi)**.

Occurrence of toxic cyanobacterial blooms (cyanoblooms) is a serious global problem which affects the water quality due to the production and accumulation of different cyanotoxins and other malodorous compounds. These blooms may cause an increase of biological oxygen demand (BOD) and anoxia in the water bodies, and death of aquatic life (Havens, 2008; Brookes and Carey, 2011; Rastogi et al., 2014). The factors contributing to the worldwide occurrence of cyanobacterial blooms are still debatable. Nevertheless, cultural eutrophication from domestic, industrial, and agricultural wastes as well as global climate change can play a major role in the global expansion of harmful algal blooms and toxin production (Kaebernick and Neilan, 2001; Conley et al., 2009; Smith and Schindler, 2009; Paerl and Scott, 2010; Kleinteich et al., 2012; O’Neil et al., 2012; Paerl and Paul, 2012; Neilan et al., 2013; Gehringer and Wannicke, 2014; **Figure 2**). Excessive loads of certain inorganic and/or organic nutrient concentrations are considered as strong risk factors for bloom promotion both in fresh and marine water habitats (Smith, 2003; Heisler et al., 2008; Conley et al., 2009; Ruttenberg and Dyhrman, 2012; Michalak et al., 2013; Davidson et al., 2014; Beversdorf et al., 2015). The anthropogenically mediated change in the N/P ratio has frequently been interrelated to the appearance of cyanobacterial blooms (Glibert et al., 2004). The phosphorus concentration was found as a primary regulating factor for increased cyanobacterial growth and changes of genotypes, both of which were found to be closely related to the water temperature, signifying the role of eutrophication in the occurrence of toxic blooms (Joung et al., 2011). Recently, Molot et al. (2014) presented a novel conceptual model linking anoxia, phosphorus (P), nitrogen (N), iron (Fe), and sulfate to the formation of harmful cyanobacterial blooms across three gradients, i.e., nutrients, salinity, and acidity. Continued transfer of sediments to a water body may block the natural flow of water and enrich the dissolved organic carbon and other compounds leading to potential risk of bloom formation.

Global climate change followed by changes in air/water temperature gradients, as well as increased nutrient precipitation can affect the cyanobacterial bloom formation and production of different cyanotoxins (Kanoshina et al., 2003; Paerl and Huisman, 2009; El-Shehawy et al., 2012; Paerl and Paul, 2012). Several environmental factors related to the dynamics of the abundance of toxic cyanobacterial bloom formation have been verified (Joung et al., 2011; Neilan et al., 2013). Warm and calm weather and low turbulence can enhance the formation of cyanobacterial blooms (Paerl and Huisman, 2008). Increased emission of ozone depleting substances (ODSs), due to huge burning of fossil-fuels and concomitant changes in air temperature, may promote the water cyanobacterial growth. As a result of climate change, the frequent droughts in summer as well as flash-flooding may lead to abandoned nutrient discharges from urban areas to unloading water bodies such as ponds, lakes, ditches, and estuaries with the consequence of the augmentation of toxic blooms and the increase of the BOD of a water reservoir (Whitehead et al., 2009). Nitrogen limitation under drought condition may cause a shift from non-N_2_-fixing to N_2_-fixing cyanobacteria leading to an increase in biologically available nitrogen and a subsequent production of cyanotoxins (Posch et al., 2012). The increased salination due to summer droughts, rising sea levels, wind flow, and common practices of the use of freshwater for agricultural irrigation, all have led to the origin and existence of several salt tolerant freshwater toxic cyanobacteria as evidenced by an increased number of blooms in brackish waters (Kanoshina et al., 2003; Paerl and Huisman, 2009). Under increased temperature and low wind mixing, the water column becomes stagnant and a large number of buoyant cyanobacteria move upward at the water surface causing dense surface blooms to fulfill their photosynthetic needs (Huisman et al., 2004; Paerl and Huisman, 2008, 2009; **Figure 2**). It has been established that dense cyanobacterial blooms require excessive CO_2_ to support their photosynthetic growth (Paerl and Huisman, 2009). Furthermore, global climate change due to anthropogenically released ODS and increased atmospheric CO_2_ levels can minimize carbon limitation of photosynthetic growth leading to increased algal biomass productions in the water reservoirs (Paerl and Huisman, 2009). Moreover, increased CO_2_ levels may increase the problems associated with the harmful cyanobacteria in eutrophic lakes (Sandrini et al., 2015). Recently, Verspagen et al. (2014) reported that rising CO_2_ levels may result in a marked intensification of phytoplankton blooms in eutrophic and hypertrophic waters. Climate change, which is predicted to lead the changes in rainfall patterns along with an increase in temperature may also influence the occurrence and severity of toxic cyanobacterial blooms due to a significant impact on inland water resources (Reichwaldt and Ghadouani, 2012). It has been suggested that UV-B radiation may significantly influence strain composition of cyanobacterial blooms in favor of microcystin (MC) producers (Ding et al., 2013). Several species/strains of bloom forming cyanobacteria produce different toxic peptides and alkaloids (**Table 1**), which are a major threat to the safe drinking water and pose a serious threat to the global environmental and human health (Kaplan et al., 2012; Rastogi et al., 2014). Until now, a number of views have been given for world-wide occurrence of cyanobacterial blooms (Paerl and Huisman, 2009; Paerl and Paul, 2012; Neilan et al., 2013; Rastogi et al., 2014); however, the exact mechanisms and the role of different environmental factors regulating the bloom dynamics are disputable and yet to be understood.

**Table 1:** Some common cyanotoxins found in different cyanobacteria and their possible toxicity and mode of actions.

Toxins	Producing cyanobacterial genera	Biological toxicity	Possible mechanisms of action	Reference
Anatoxin a-(s)	*Anabaena*	Neurotoxic	Inhibition of Ach-esterase activity, hyper-excitability of nerve	Matsunaga et al., 1989
Anatoxin-a	*Anabaena, Aphanizomenon, Cylindrospermum, Microcystis, Planktothrix, Raphidiopsis*	Neurotoxic	Depolarizing neuromuscular blocking	Devlin et al., 1977; Carmichael, 1998
Antillatoxin	*Lyngbya*	Neurotoxic	Blocking neuronal communication by binding to the voltage-gated Na^+^ channels	Berman et al., 1999; Li et al., 2001
Aplysiatoxins	*Lyngbia, Schizothrix, Trichodesmium, Oscillatoria*	Dermatotoxic	Potent tumor promoters and protein kinase C activators	Fujiki et al., 1982
Cylindrospermopsin	*Anabaena, Aphanizomenon Cylindrospermopsis, Lyngbya, Oscillatoria* (*Planktothrix*), *Rhaphidiopsis, Umezakia*	Hepatotoxic, nephrotoxic, and cytotoxic	Irreversible inhibition of protein and glutathione synthesis, implicating cytochrome P-450, overexpression of DNA damage repair proteins	Humpage et al., 2000; Froscio et al., 2003; Neumann et al., 2007
Cyanopeptolin	*Microcystis, Planktothrix*	Neurotoxic activity	Transcriptional alterations of genes belonging to DNA damage and repair	Faltermann et al., 2014
Homoanatoxin-a	*Anabaena, Oscillatoria* (*Planktothrix*), *Phormidium, Raphidiopsis*	Neurotoxic	Blockade of the neuromuscular transmission	Aas et al., 1996; Lilleheil et al., 1997
Jamaicamides	*Lyngbya*	Neurotoxic, cytotoxic	Blocking voltage-gated sodium channels	Edwards et al., 2004
Kalkitoxin	*Lyngbya*	Neurotoxic	Blocking voltage-gated sodium channels	Wu et al., 2000; LePage et al., 2005
Lipopolysaccharides (LPS)	*Anabaena, Anacystis, Microcystis, Oscillatoria, Spirulina*, and almost all cyanobacteria	Dermatotoxic	Impairment of immune and detoxification system, irritation, and allergic effects	Mankiewicz et al., 2003; Wiegand and Pflugmacher, 2005
Lyngbyatoxin-a	*Lyngbya, Oscillatoria, Schizothrix*	Cytotoxic, dermatotoxic, gastroenteritis	Dermonecrotic, protein kinase C activator, and potent tumor promoters	Cardellina et al., 1979; Fujiki et al., 1981, 1984
Microcystins	*Anabaena, Anabaenopsis, Aphanocapsa, Aphanizomenon, Arthrospira, Cyanobium, Cylindrospermopsis, Fischerella, Hapalosiphon, Limnothrix, Lyngbya, Microcystis, Nostoc, Oscillatoria* (*Planktothrix*), *Phormidium, Planktothrix, Rivularia, Synechocystis*, and *Synechococcus*	Hepatotoxic	Inhibitors of protein phosphatases 1, 2A and 3, tumor promoter, genotoxicity	Honkanen et al., 1990; MacKintosh et al., 1990; Gulledge et al., 2002
Nodularins	*Nodularia*	Hepatotoxic	Inhibitors of protein phosphatases 1, 2A and 3, tumor promoter	Yoshizawa et al., 1990; Gulledge et al., 2002
Saxitoxins	*Anabaena, Aphanizomenon, Cylindrospermopsis, Lyngbya, Planktothrix, Raphidiopsis, Scytonema*	Neurotoxic	Blocking neuronal communication by binding to the voltage-gated Na^+^ channels	Strichartz et al., 1986; Su et al., 2004
β-*N*-methylamino-L-alanine (BMAA)	*Anabaena, Microcystis, Nostoc, Planktothrix*	Neurotoxic	Motor system disorder, glutamate agonist, increasing the intracellular concentration of calcium in neurons and inducing neuronal activity by hyperexcitation	Brownson et al., 2002; Lobner et al., 2007

Our understanding of the responses of various environmental factors associated with climate change and their impact on marine/freshwater ecosystems is based on several experimental and/or inferential data. From the above discussions, it is clear that the appearance of a cyanobacterial bloom is the consequence of several coherent signals. It is utmost important to unravel the specific effects of nutrient enrichment and other global climate change on our aquatic ecosystem, and to establish the facts on how the structure and function of an ecosystem can be maintained. Moreover, if the existing level of anthropogenically induced nutrient loading in the water bodies and environmental warming continues, multiple-fold increase in algal bloom followed by contamination of our aquatic ecosystem by several toxic substances is expected in future. Henceforth, most conceptual and empirical research on the triggers of cyanobacterial blooms is needed to understand the multifarious set of situations that influence the worldwide incidence of toxic cyanoblooms.

## Toxins From Cyanobacteria

Cyanobacteria produce a wide range of toxic secondary compounds causing human and domestic/wildlife intoxication. A number of bloom forming cyanobacteria from diverse habitats have been reported to produce different toxins (Rastogi et al., 2014). Chemically, the cyanotoxins are divided into three main groups, i.e., cyclic peptides (MCs and nodularins), alkaloids (anatoxin-a, anatoxin-a(s), saxitoxins, cylindrospermopsin, aplysiatoxin, lyngbiatoxin-a), and lipopolysaccharides (LPSs; Kaebernick and Neilan, 2001). However, based on biological effects, the cyanobacterial toxins can be classified into five functional groups such as hepatotoxins, neurotoxins, cytotoxins, dermatotoxins, and irritant toxins (Sivonen and Jones, 1999; Codd et al., 2005).

## Cyclic Peptides

**FIGURE 3:**
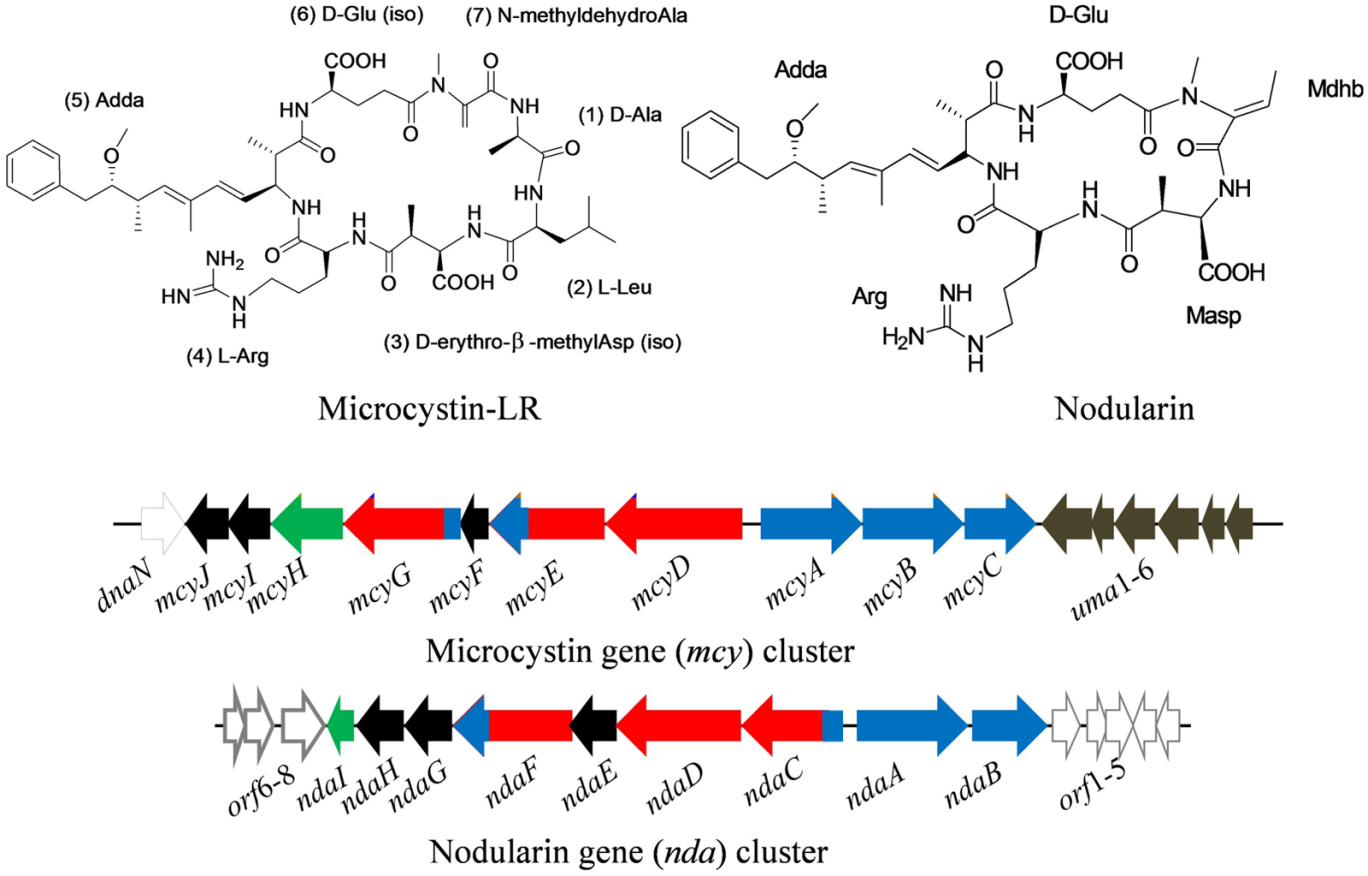
**Chemical structure of microcystin (MC-LR) and nodularin (NOD), and their biosynthetic gene clusters, mcy and *nda* in the cyanobacteria *Microcystis aeruginosa* PCC7806 and *Nodularia spumigena* NSOR10, respectively**.

Among the different cyanobacterial toxins, MCs are the most frequently occurring cyanotoxins in surface as well as drinking water and widely investigated hepatotoxins. MCs are cyclic heptapeptides (**Figure 3**) produced by several strains of cyanobacteria (Sivonen and Jones, 1999; Krienitz et al., 2002; Izaguirre et al., 2007; Aboal and Puig, 2009; Rastogi et al., 2014; **Table 1**). Currently, more than 90 variants of MCs are known, all with the general structure cyclo-(D-Ala-X-D-MeAsp-Z-Adda- D-Glu- Mdha), X and Z being variable L-amino acids. On the basis of acute toxicity, microcystin-LR (MC-LR) is considered the most potent hepatotoxin (Funari and Testai, 2008).

Black – tailoring enzymes, red – polyketide synthases, blue – non-ribosomal peptide synthetases, light black – non-microcystin synthetase, green – ABC transporter (adapted from Tillett et al., 2000; Moffitt and Neilan, 2004; Gehringer et al., 2012; Gehringer and Wannicke, 2014; Gene cluster not drawn to scale).

Microcystin is synthesized non-ribosomally by large multi-enzyme complexes comprising different modules including non-ribosomal peptide synthetases (NRPSs) as well as polyketide synthases (PKSs), and several tailoring enzymes. The gene cluster responsible for MC biosynthesis has been identified in different cyanobacteria (Tillett et al., 2000; Rouhiainen et al., 2004; Christiansen et al., 2008; Gehringer et al., 2012). In the cyanobacterium *Microcystis aeruginosa* PCC7806, the MC gene clusters spans 55 kb of DNA and is composed of 10 (mcyABCDEFGHIJ) bidirectionally transcribed open reading frames (ORFs) arranged in two divergently transcribed operons, *mcyA-C* and *mcyD-J* (Tillett et al., 2000; **Figure 3**). The assembly of MC begins with the activation of a phenylalanine-derived phenyl propionate starter unit at the NRPS/PKS hybrid enzyme McyG (Hicks et al., 2006). The gene clusters encoding MC biosynthesis sequence from the *Microcystis* (Tillett et al., 2000), *Planktothrix* (Christiansen et al., 2008), and *Anabaena* (Rouhiainen et al., 2004) species revealed that the arrangements of ORFs in the *mcy* cluster vary among different genera. However, a high sequence similarity between the *mcy* gene clusters of different genera suggests a common ancestor for MC synthesis (Rantala et al., 2004).

Similar to MCs, cyclic pentapeptide toxic compounds, nodularins (NODs; **Figure 3**) represent the second group of hepatotoxins produced by the cyanobacteria *Nodularia* and *Nostoc.* At present, more than seven variants of NOD have been reported. Both hepatotoxins (MCs and NODs) contain a unique hydrophobic amino acid, Adda (2S,3S,8S,9S-3-amino-9-methoxy-2,6,8-trimethyl-10-phenyl-deca-4,6-dienoic acid). Chemically, NODs differ from MCs in terms of the absence of two core amino acids and have *N*-methyldehydrobutyrine (*Mdhb*) instead of *N*-methyldehydroalanine (*Mdha*; Rinehart et al., 1998). Similar to MCs, NODs are also produced non-ribosomally from *nda* gene clusters by means of NRPS-PKS enzyme systems (Moffitt and Neilan, 2004; **Figure 3**). In the cyanobacterium *Nodularia spumigena* NSOR10, the locus of *nda* gene clusters (48 kb) consists of nine ORFs (*ndaA–I*) transcribed from a bidirectional regulatory promoter region (Moffitt and Neilan, 2004). Moreover, MCs and NODs show similar biological activity in spite of their different chemical structures. These cyclic peptides inhibit the specific protein serine/threonine phosphatases-1 (PP1) and -2A (PP2A) which are important regulatory enzymes in eukaryotic cells (MacKintosh et al., 1990).

## Alkaloids

**FIGURE 4:**
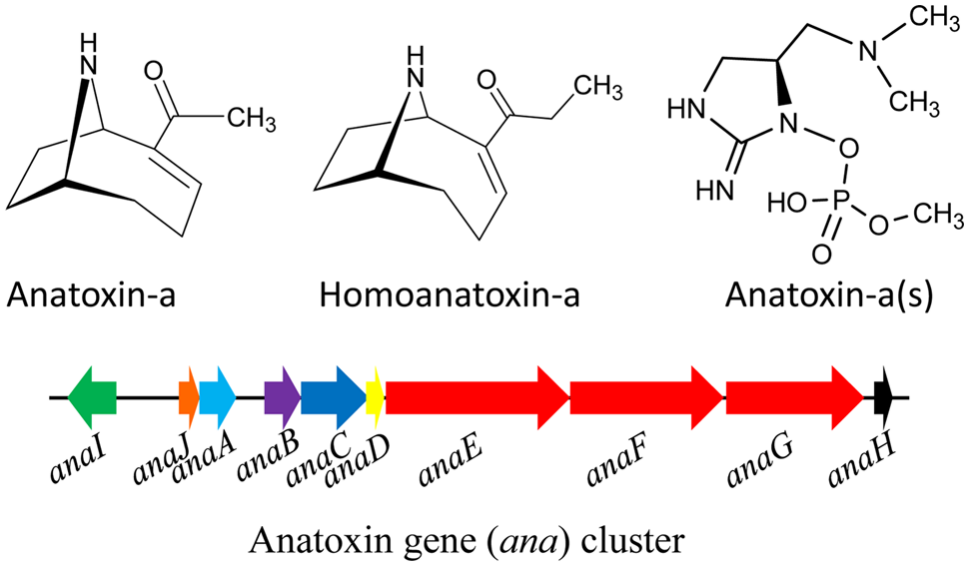
**Chemical structure of anatoxins and its biosynthetic gene (*ana*) cluster in the cyanobacterium *Oscillatoria* sp. PCC6506.** Green – transporter, orange – cyclase, light blue – thioesterase, purple – oxidase, blue- adenylation protein, yellow – acyl carrier protein, red – polyketide synthase, black – transposase (adapted from Rantala-Ylinen et al., 2011; Méjean et al., 2014; Gene cluster not drawn to scale).

A number of toxic alkaloids have been found in different cyanobacteria. The alkaloids anatoxin-a (MW = 165 Da) and its homolog homoanatoxin-a (MW = 179 Da) are fast-acting neurotoxins, also known as fast death factors (FDFs). Anatoxin-a (**Figure 4**) was first isolated from *Anabaena flos-aquae* and so far has been found in several cyanobacteria such as *A. circinalis, A. planctonica, A. spiroides, Aphanizomenon, Cylindrospermum, Planktothrix*, and *M. aeruginosa* (Edwards et al., 1992; Park et al., 1993; **Table 1**). The alkaloid homoanatoxin-a has a methylene group at C-2 instead of the acetyl group (**Figure 4**) and structurally resembles anatoxin-a. Homoanatoxin-a has been isolated from the cyanobacteria *Oscillatoria* (*Planktothrix*) *formosa, Phormidium formosum, Anabaena*, and *Raphidiopsis mediterranea* (Furey et al., 2003; Namikoshi et al., 2003; Watanabe et al., 2003). Another homolog of anatoxin, anatoxin-a(s) (MW 252 Da; **Figure 4**), isolated from *A. flos-aquae* and *A. lemmermannii*, is a potent acetylcholinesterase (AChE) inhibitor (Matsunaga et al., 1989) but more lethal than anatoxin-a (Carmichael et al., 1990; Méjean et al., 2014). It is synthesized in the cell from ornithine *via* putrescine catalyzed by the enzyme ornithine decarboxylase. Moreover, the partial genome sequencing demonstrated the presence of putative gene cluster (Méjean et al., 2014) encoding the biosynthetic pathway of anatoxin-a and homoanatoxin-a in cyanobacteria such as *Oscillatoria* PCC 6506 (Méjean et al., 2009) and *Anabaena* strain 37 (Rantala-Ylinen et al., 2011; **Figure 4**).

**FIGURE 5:**
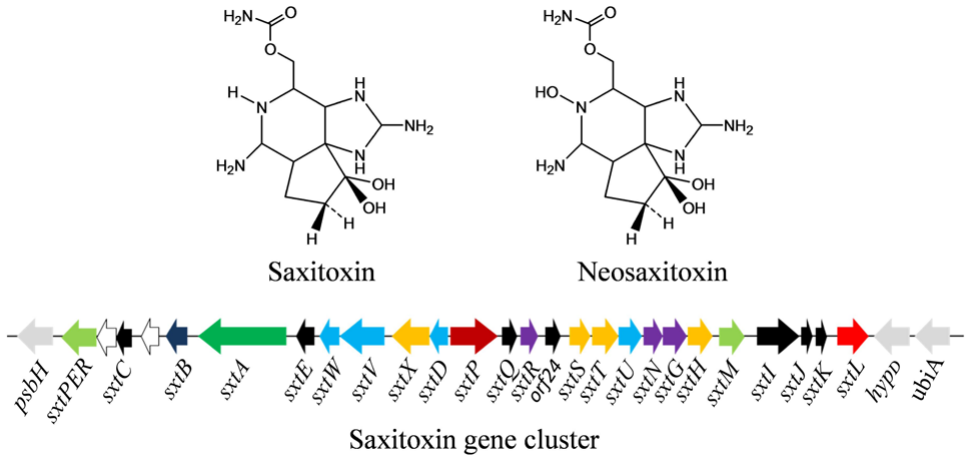
**Chemical structure of saxitoxin and its biosynthetic gene cluster in the cyanobacterium *Aphanizomenon* sp. NH-5.** Light green – transporter, white – transposase, black – unknown, dark blue – cyclase, green – polyketide synthase, light blue – oxido-reductase, orange – hydroxylase, dark red – putative regulator, orange – transferase, red – hydrolase (for details, see Kellmann et al., 2008; Mihali et al., 2009; Gene cluster not drawn to scale).

Saxitoxin and its analogs (e.g., neosaxitoxin; **Figure 5**) are a group of carbamate alkaloid toxins which all are highly potent neurotoxins. These are tricyclic compounds, consisting of a tetrahydropurine group and two guanidine subunits, commonly called paralytic shellfish poisons (PSPs). Currently, about 27 variants of saxitoxins have been found in different cyanobacteria such as *Aphanizomenon, Anabaena flos-aquae, Anabaena circinalis, Lyngbya wollei*, and *Cylindrospermopsis raciborskii* (**Table 1**). Regulation of saxitoxin biosynthetic pathway and characterization of some enzymes involved are not well-studied (Soto-Liebe et al., 2010). However, it has been postulated that biosynthesis of saxitoxin depends on the multifunctional PKS enzyme, SxtA (Kellmann et al., 2008). The saxitoxin biosynthetic gene cluster (25.7–36 kb) includes 33 genes, reported in cyanobacteria such as *Cylindrospermopsis raciborskii* (strain T3), *Anabaena circinalis* (strain AWQC131C), *Aphanizomenon* strain NH-5, *Lyngbya wollei*, and *Raphidiopsis brookii* (strain D9; Kellmann et al., 2008; Mihali et al., 2009, 2011; Soto-Liebe et al., 2010; Stucken et al., 2010; Neilan et al., 2013; **Figure 5**). The positions of genes encoding biosynthetic enzymes, transporters, and regulatory proteins within the cluster differ among the different cyanobacterial strains dicsussed above. Moreover, the toxic profile expressed in different strains is determined by the position and presence, or absence, of specific genes in the respective clusters.

**FIGURE 6:**
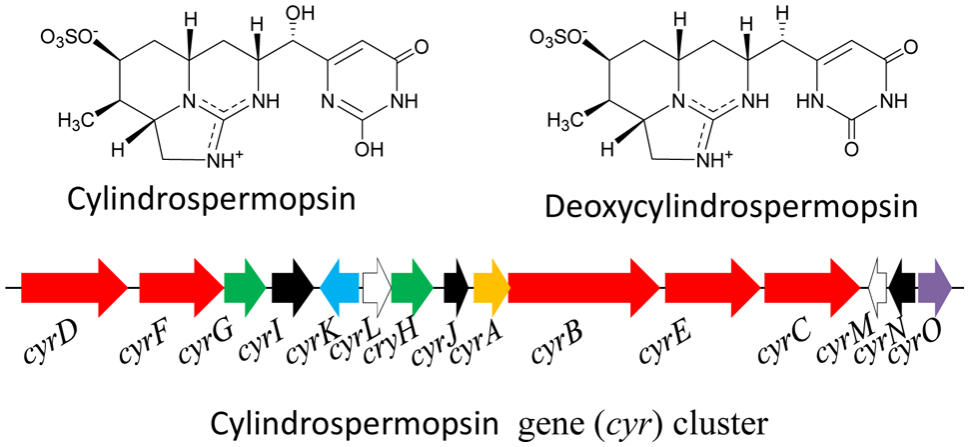
**Chemical structure of cylindrospermopsin and its biosynthetic gene (*cyr*) cluster in the cyanobacterium *Cylindrospermopsis raciborskii* AWT205.** Red – PKS/NRPS, green – uracil ring, black – tailoring, blue – transport, white – transposase, orange – amidinotransferase, purple – regulator (Adapted from Mihali et al., 2008).

The cyanotoxin cylindrospermopsin (CYN: MW 415 Da) is a polyketide-alkaloid having a tricyclic guanidine moiety and sulfate groups (**Figure 6**). Presently, some analogs of CYN such as deoxy-cylindrospermopsin, demethoxy-cylindrospermopsin and 7-epicylindrospermopsin have been identified in the cyanobacteria *C. raciborskii* (Norris et al., 1999) and *Aphanizomenon ovalisporum* (Banker et al., 2000). The CYN variant 7-epicylindrospermopsin differs due to the orientation of the hydroxyl group close to the uracil moiety (Banker et al., 2000), and the other variant deoxy-cylindrospermopsin is characterized by a missing oxygen atom related to the initial hydroxyl group close to uracil moiety. Moreover, a number of cyanobacteria such as *Cylindrospermopsis raciborskii, Aphanizomenon ovalisporum, Aphanizomenon flos-aquae, Anabaena lapponica, Anabaena bergii, Lyngbya wollei, Umezakia natans, Raphidiopsis curvata*, and *Oscillatoria* (*Planktothrix*) have been reported to produce CYN and its analogs (Ohtani et al., 1992; Harada et al., 1994; Banker et al., 1997; Preussel et al., 2006; Spoof et al., 2006; Seifert et al., 2007; Mazmouz et al., 2010). McGregor et al. (2011) reported the presence of the cyanotoxins CYN and deoxy-CYN from the cyanobacterium *Raphidiopsis mediterranea* FSS1-150/1 of a eutrophic reservoir in Queensland, Australia. CYN shows hepatotoxic, nephrotoxic, and cytotoxic effects and is a potential carcinogen owing to the inhibition of glutathione, cytochrome P450 and protein synthesis (Humpage et al., 2000; Froscio et al., 2003; Neumann et al., 2007). The gene cluster (*cyr*) encoding the enzymes of the CYN biosynthesis (**Figure 6**) has been reported to be present in several cyanobacteria such as *C. raciborskii* (Mihali et al., 2008; Stucken et al., 2010; Jiang et al., 2012), *Aphanizomenon* strain 10E6 (Stuken and Jakobsen, 2010), and *Oscillatoria* PCC 6506 (Mazmouz et al., 2010). The arrangements of genes and flanking regions differ across genera; however, all the gene clusters are highly conserved with respect to the nucleotide sequence of orthologous genes (Neilan et al., 2013). In case of the cyanobacterium *C. raciborskii* AWT205, the *cyr* gene cluster (42 kb) encodes 15 ORFs (*cyrA*-O). The biosynthesis of CYN is initiated by an amidinotransferase and completed by NRPS-PKS-type enzymes in combination with tailoring enzymes (Muenchhoff et al., 2010). As stated above, the gene cluster for CYN biosynthesis has been sequenced from several cyanobacteria; however, few studies have been conducted on its transcriptional organization and promoter structure (Stuken and Jakobsen, 2010).

## Lipopolysaccharides

The endotoxins LPSs consist of an internal acylated glycolipid (lipid-A), core domain (an oligosaccharide) and an outer polysaccharide (O-antigen) chain (Raetz and Whitfield, 2002). In general, the fatty acid component (lipid-A) of LPS is responsible for the toxic actions such as irritant and allergenic responses in human and animal tissues (Mankiewicz et al., 2003). The LPSs present in cyanobacteria differ from those in enteric bacteria by having a larger variety of long chain unsaturated fatty acids and hydroxy fatty acids and the lack of phosphate. Moreover, there is substantial diversity of LPSs composition among the cyanobacteria, although variations are basically related to phylogeny. Different genera of cyanobacteria have distinct LPSs compositions conserved within the particular genus (Sivonen and Jones, 1999). Several cyanobacteria such as *Anacystis nidulans, Microcystis, Anabaena, Spirulina*, and *Oscillatoria* all have been reported to produce LPS toxin (Smith et al., 2008; Bláhová et al., 2013). The structure of the lipid-A subunit in the cyanobacterial LPS molecule has not been clearly identified, and furthermore, the exact mechanism of LPS toxicity produced by cyanobacteria is still unknown.

**FIGURE 7:**
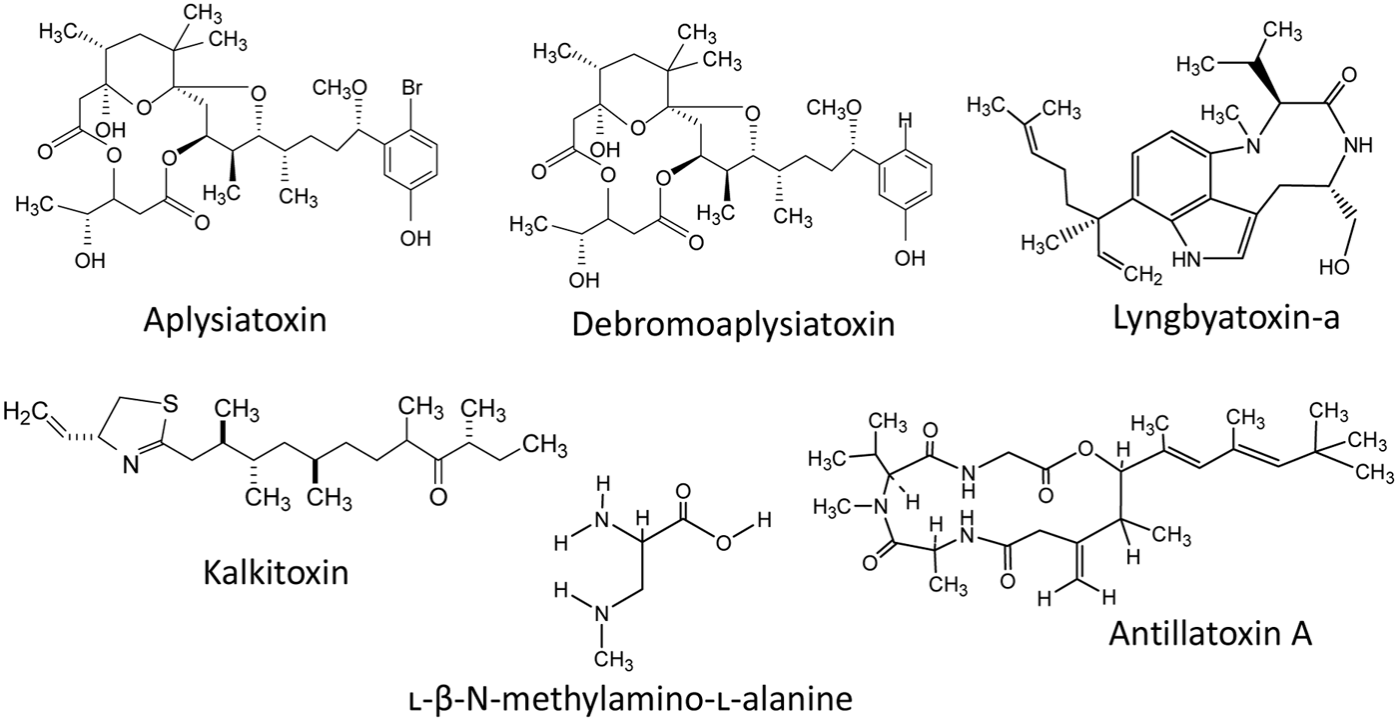
**The chemical structure of some common cyanotoxins reported in diverse cyanobacteria**.

Besides the above mentioned cyanotoxins, a number of toxins such as aplysiatoxin, kalkitoxin, antillatoxin, lyngbyatoxins, cyanopeptolin, aurilides, and jamaicamides have been reported to be present in different cyanobacteria in fresh and/or marine water habitats (**Figure 7**). A phenolic bislactone alkaloid aplysiatoxin has been reported from several cyanobacteria such as *Lyngbia majuscula, Schizothrix calcicola, Trichodesmium erythraeum*, and *Oscillatoria nigroviridis* (Mynderse et al., 1977; Gupta et al., 2014). Aplysiatoxin and debromoaplysiatoxin (**Figure 7**) are potent tumor promoters and protein kinase C activators and show signs of several lethal effects. Moreover, an analog of the tumor-promoting aplysiatoxin has been reported as an antineoplastic agent rather than a tumor-promoting substance (Nakagawa et al., 2009). Recently, the analogs of aplysiatoxin debromoaplysiatoxin and anhydrodebromoaplysiatoxin, as well as two new analogs, 3-methoxyaplysiatoxin and 3-methoxydebromoaplysiatoxin have been reported from the marine cyanobacterium *Trichodesmium erythraeum* (Gupta et al., 2014). The alkaloid lyngbyatoxin, a prenylated cyclic dipeptide compound, was isolated from *Lyngbya majuscula* (Taylor et al., 2014) and has several similarities with aplysiatoxin in its mechanism of toxicity and both are potent tumor promoters. Kalkitoxin (**Figure 7**) is a lipopeptide neurotoxin produced by some species of cyanobacteria such as *L. majuscula* (Berman et al., 1999). The antillatoxin is an ichthyotoxic cyclic depsipeptide isolated from *L. majuscula* (Orjala et al., 1995). A number of bioactive peptides such as microviridins, microginins, cyanopeptolides, and β-*N*-methylamino-L-alanine (BMAA; **Figure 7**) have also been reported from diverse cyanobacteria, but their toxicological profiles and impacts on the environment as well as human health are not known (Downing et al., 2014). Moreover, the cyanobacterial neurotoxin, BMAA has been suggested to function as a causative agent for certain neurodegenerative diseases (Lobner et al., 2007). The compound curacin-A, isolated from *L. majuscula* (Gerwick et al., 1994), exhibited potent anti-proliferative and cytotoxic activity against colon, renal, and breast cancer derived cell lines (Verdier-Pinard et al., 1998). A cyanobacterial toxin cyanopeptolin (CP1020) produced by *Microcystis* and *Planktothrix* strains was found to cause transcriptional alterations of genes involved in DNA damage and repair (Faltermann et al., 2014). Recently, two new cyanobacterial peptides named micropeptins 1106 and 1120 were reported from cyanobacterial blooms in North Carolina’s Cape Fear River. However, their biological activities have not yet been determined (Isaacs et al., 2014). Moreover, several studies indicate the presence of several additional, still unidentified and not characterized biotoxins in cyanobacterial blooms.

## Ecological Health Impacts of Cyanotoxins

The increased incidence of toxic cyanobacterial blooms is posing potential risks to aquatic ecosystem as well as human and animal health. Cyanotoxins may cause several harmful effects on humans or animals either through direct contact or by means of intake of untreated contaminated water and food (Miller et al., 2010; Papadimitriou et al., 2012; Rastogi et al., 2014; Sukenik et al., 2015). Aquatic organisms may be affected either through direct ingestion of toxic cyanobacterial cells or through contact with cyanotoxins. It has been established that intake of contaminated water or food is a key route for cyanotoxin intoxication (Zhang et al., 2009; Miller et al., 2010). Several secondary compounds have been reported to have their toxic effects on different organisms ranging from plant to animals. In the subsequent section we have focused on the adverse toxic effects of some common cyanotoxins on aquatic/wild animals and humans.

The cyanotoxin MCs are well-known for their toxic effects. MCs can affect the cellular system through disorganization of cytoskeleton, cell proliferation, genome damage, inhibition of enzyme activity, imprecise mitotic cell division, loss of membrane integrity, oxidative stress, and lipid peroxidation (Rastogi et al., 2014). To know the detailed mechanisms or mode of action of MCs, readers are referred to the recent review by Rastogi et al. (2014). MCs act by blocking protein PP1 and -2A, causing toxicity at the hepatic level. It has been demonstrated that MC-LR can induce reproductive (Chen et al., 2011; Zhou et al., 2012) as well as cardio-toxicity in animals (Qiu et al., 2009). MC-LR was found to cause normocyte anemia and the bone marrow injury, and also affected the immune system of rabbits (Zhang et al., 2011; Yuan et al., 2012). Moreover, a number of fatal poisonings of MCs regarding the health risk of domestic and wild animals, birds, fish, and several other aquatic as well as terrestrial organisms have been reported worldwide (Stewart et al., 2008; Rastogi et al., 2014). The mass mortalities of Lesser Flamingos were reported at Lake Bogoria, Kenya due to MC intoxication (Krienitz et al., 2003). A new episode of cyanotoxin (MC-LR, -YR, and -RR) intoxication and mass mortalities of Lesser Flamingos (*Phoeniconaias minor* Geoffroy) have also been reported at Lake Manyara in Tanzania (Nonga et al., 2011). At least 6,000 birds belonging to 47 species, including endangered species such as the marbled teal (*Marmaronetta angustirostris*) and white-headed duck (*Oxyura leucocephala*), died due to MC-LR intoxication at the Doñana National Park, Spain (Lopez-Rodas et al., 2008).

Despite numerous reports of cyanotoxins impact on the aquatic organisms and wild or domestic animals, the epidemiological facts for cyanotoxins intoxication in humans are very limited (Rastogi et al., 2014). Recent studies have established the cytotoxic and genotoxic potentials of various cyanotoxins including MCs (Žegura et al., 2011). The use of untreated water contaminated with cyanobacterial blooms and MCs resulted in normocytic anemia (Pouria et al., 1998), liver failure and several other symptoms such as nausea, vomiting, and acute liver damage leading to human death in a hemodialysis center in Caruaru, Brazil (Pouria et al., 1998; Hilborn et al., 2007). The use of MC-contaminated water can be a potential risk factor for liver and colorectal cancer among humans (Lun et al., 2002; Hernández et al., 2009). Moreover, MCs may cause hepatotoxicity and neurotoxicity, kidney impairment, allergies and eye, ear and skin irritation, and certain gastrointestinal disorders such as nausea/vomiting and diarrhea in humans (Torokne et al., 2001; Pilotto et al., 2004; Codd et al., 2005).

As stated above, the cyanotoxin NODs have chemical structure as well as mechanisms of action similar to those of MCs (Yoshizawa et al., 1990); however, NODs have not been studied as extensively as MCs (Funari and Testai, 2008). NODs are a potent inhibitor of protein phosphatase 1 and 2A (Ohta et al., 1994) and show accumulative toxicity and tumor formation (Ohta et al., 1994; Sueoka et al., 1997; Song et al., 1999). The toxic effects of NODs have also been investigated in fish (Sotton et al., 2015). In the flatfish *Platichthys flesus*, NODs induced oxidative stress as indicated by a decrease of GST and CAT activity resulting in increased vulnerability of the cells to reactive oxygen species (ROS; Persson et al., 2009). NOD can also induce apoptosis and hyperphosphorylation of signaling proteins in cultured rat hepatocytes (Ufelmann and Schrenk, 2015). Nevertheless, not much toxicological data are available for NODs carcinogenicity in humans.

A cytotoxic alkaloid CYN can irreversibly inhibit the biosynthesis of protein and glutathione leading to cell death (Ohtani et al., 1992; Terao et al., 1994; Froscio et al., 2003). A *Cylindrospermopsis* bloom episode was found to cause cattle mortalities and human poisonings in north Queensland (Saker et al., 1999; Griffiths and Saker, 2003). Moreover, a number of disorders such as damage to liver, kidney, thymus, and heart, as well as hepatic and renal toxicity were observed in mice (Terao et al., 1994; Falconer et al., 1999; Bernard et al., 2003; Froscio et al., 2003). CYN may induce DNA strand breaks and possibly disrupt the kinetochore spindle, leading to chromosome loss, specifying its clastogenic and aneugenic action (Humpage et al., 2000). In primary rat hepatocytes, CYN has been shown to inhibit protein and glutathione synthesis and induce apoptosis (López-Alonso et al., 2013). Recently, Huguet et al. (2014) studied the effects of CYN on human intestinal Caco-2 cells and reported that CYN can modulate different biological functions by overexpressing the genes encoding proteins involved in DNA damage repair and transcription including modifications of nucleosomal histones. It has been shown that CYN may cause a decrease in glutathione synthesis (Runnegar et al., 1994) and induce oxidative stress in fish (Guzmán-Guillén et al., 2013a,b). Indeed, CYN can accumulate in various organs of fish, leading to deleterious effects on their normal physiology and biochemistry (Sotton et al., 2015). CYN may interfere with the basic functions of fish phagocytic cells and as a consequence, influence the fish immunity (Sieroslawska and Rymuszka, 2015).

A number of neurotoxic alkaloids from cyanobacteria have been reported, exerting their action on the neuromuscular system by blocking skeletal and respiratory muscles leading to respiratory failure. The cyanotoxins such as anatoxins, saxitoxins, antillatoxin, kalkitoxin, and jamaicamide are major groups of neurotoxic compounds (Aráoz et al., 2010). It has been established that anatoxin-a is a potent depolarizing neuromuscular blocking agent which acts by binding to nicotinic receptors for acetylcholine in the central nervous system (CNS), peripheral nervous system (PNS) and in neuromuscular junctions (Carmichael, 1998). Several studies regarding the mechanisms of anatoxins toxicity were performed in mice using the sub-lethal or lethal doses of anatoxin-a. Anatoxin-a, well-known as a “Very Fast Death Factor,” can cause contraction, muscular paralysis, and respiratory arrest leading to death of mice in a very short time after intraperitoneal injection (i.p. mouse LD_50_: 250 to 375 μg/kg; Devlin et al., 1977). Anatoxin-a can impair blood pressure, heart rate and gas exchange triggering hypoxia, respiratory arrest and severe acidosis leading to death of the animals (Adeymo and Sirén, 1992). The toxicological properties of homoanatoxin-a are more or less identical to those of anatoxin-a (Namikoshi et al., 2003). The neurotoxic alkaloid saxitoxins are considered the most toxic compounds. The mode of action of all analogs of saxitoxins is more or less similar; however, they differ in toxicity (Funari and Testai, 2008). Saxitoxins may block voltage-gated sodium channels in nerve cells and discontinue the entry of sodium flow, preventing the generation of a proper action potential or electrical transmission in nerves and muscle fibers leading to paralysis of muscles and death by respiratory arrest in mammals (Strichartz et al., 1986; Su et al., 2004; Bricelj et al., 2005). Another neurotoxic cyanotoxin antillatoxin is a novel ichthyotoxic (LC_50_ = 0.1 μM) cyclic lipopeptide isolated from the marine cyanobacterium *Lyngbya majuscula* (Orjala et al., 1995). Antillatoxin-A prompted a rapid neuronal death in cerebellar granule cell cultures (LC_50_ = 0.18 μM; Berman et al., 1999). Voltage-gated sodium channels were shown as the main molecular target of antillatoxin (Li et al., 2001). The neurotoxic compound kalkitoxin isolated from *L. majuscula* is a thiazoline-containing lipopeptide compound (Wu et al., 2000). Lyngbyatoxin-A, a cyclic dipeptide found in *L. majuscula*, appears to have been responsible for a severe oral and gastrointestinal inflammation suffered by a person who accidentally ingested this cyanobacterium (Sims and Zandee Van Rillaud, 1981). Kalkitoxin was shown ichthyotoxic to the goldfish *Carassius auratus* and toxic to the aquatic crustacean brine shrimp (*Artemia salina*) with an LC_50_ 700 and 170 nM, respectively (Wu et al., 2000). Kalkitoxin may also block voltage-gated sodium channels (LePage et al., 2005). The neurotoxic amino acid BMAA acts in mammals as a glutamate agonist (Corbel et al., 2014). BMAA increases the intracellular concentration of calcium in neurons and induces neuronal activity by hyperexcitation (Brownson et al., 2002).

The endotoxic LPSs are known to cause fever in mammals and are involved in septic shock syndrome and liver injury (Choi and Kim, 1998). LPS can impair the immune system and also affect the detoxification system of diverse organisms (Wiegand and Pflugmacher, 2005). Until now, very little is known about the LPS intoxication and its toxicity is assumed to be associated with the host-mediated factors (Stewart et al., 2006a,b). More extensive research is needed to clarify a definite toxicity mechanism of LPS. Overall, it is no doubt that the acute effects of several cyanotoxins represent the major concern for ecological health impacts.

## Cyanoblooms and Cyanotoxins: Mitigation Strategies

The increased incidence of toxic cyanobacterial blooms (cyanoblooms) worldwide and their potential health risks have generated tremendous concern for dynamic management of toxic cyanoblooms. The economic cost of freshwater blooms in the United States was estimated to be about 2.2–4.6 billion dollars/annum (Dodds et al., 2009). Henceforth, advanced approaches or development of a new technology is needed to terminate or prevent/suppress the harmful cyanobacterial blooms for environmental sustainability and economic vitality (Hudnell, 2008, 2010; Srivastava et al., 2013; Harris et al., 2014; Koreivienë et al., 2014). Several factors boosting the incidence of harmful cyanobacterial blooms, such as nutrient input, wind velocity, sediment deposition, reduced water flow, increased salinity and temperature gradients, global warming and drought can be regulated to a certain extent to eliminate or minimize the bloom incidence. The approaches implemented for bloom suppression should be environmentally sustainable without adversely influencing the aquatic ecosystems. A number of strategies or approaches such as chemical, physical, biological, and other cognizance approaches came into consideration for mitigating the harmful cyanobacterial bloom incidences.

## Chemical Approaches

Cyanoblooms can be controlled to a certain extent using some chemicals such as algicides, inhibitors or flocculants; however, use of these chemicals can inevitably recontaminate water bodies (Murray-Gulde et al., 2002; Van Hullebusch et al., 2002; Jančula and Maršálek, 2011). The use of certain pigments (aquashade) can reduce the amount of light availability, and inhibit the growth of harmful algae; however, this approach may not be effective due to growth inhibition of other beneficial microalgae, thereby undesirably influencing the aquatic ecosystems (Spencer, 1984). The use of some algicides has been reported to decline the bloom formation. The natural product cyanobacterin has been shown to be toxic to most cyanobacteria at a concentration of approximately 5 μM (Gleason and Baxa, 1986). Many biologically derived (but non-antibiotic) bioactive substances are known to inhibit the growth of aquatic bloom-forming cyanobacteria (Shao et al., 2013). Recently, Dai et al. (2012) have shown the fast removal (up to 98.99%) of MC-LR by a low-cytotoxic microgel- Fe(III) complex. Preoxidation with chlorine dioxide followed by flocculation and settling was found effective in removing cyanobacterial blooms and MCs (Bogialli et al., 2013). The use of aluminum salts can be used as algicides for nuisance algae and cyanobacteria control (Lelkova et al., 2008). The use of slaked lime [Ca(OH)_2_] or calcite (CaCO_3_) has also been reported to remove the algal communities, including cyanobacteria (Prepas et al., 2001; Zhang et al., 2001). Aluminum compounds can be used to remove the nutrients from industrial and domestic wastewaters (Auvray et al., 2006; Rodriguez et al., 2008; De Julio et al., 2010). Besides aluminum, several other metals such as iron and copper are used to remove the algal blooms. The salt of copper (CuSO_4_.5H_2_O) is widely used as an algicide (Murray-Gulde et al., 2002). The herbicide diuron together with copper sulfate as well as other copper-based compounds have been approved by the United States Environmental Protection Agency (USEPA) for use as algicides in fish production ponds (Schrader et al., 2004). Moreover, the use of synthetic compounds for bloom control has their own limitations, and therefore, a range of natural chemicals (e.g., anthraquinone, nostocarboline, and stilbenes) from diverse organisms have been derived as potent substituents of synthetic algicides (Schrader et al., 2003, 2004; Becher et al., 2005; Mizuno et al., 2008; **Table 2**). Recently, Jančula and Maršálek (2011) reviewed the availability of different chemical compounds for prevention and management of cyanobacterial blooms.

**Table 2:** Allelochemicals and their inhibitory effects against some bloom forming cyanobacteria.

Allelochemicals	Source	Target cyanobacteria	EC_50_	Mechanisms	Reference
(+)-catechin	*Myriophyllum spicatum*	*M. aeruginosa*	5.5 mg l^-1^	Growth inhibition, produced radicals	Nakai et al., 2000
1-Desgalloyleugeniin	*Myriophyllum bradieme*	*M. atwginosa, Anabaena flos-aquae*	3.7 μM	Growth inhibitory activity	Saito et al., 1989
3-oxo-a-ionone	Periphyton biofilm	*Microsystis aeruginosa*	–	Thylakoid membrane damage, failure of photosynthesis	Wu et al., 2011
4-OH-coumarin	*Ruta graveolens*	*Synechococcus leopolensis, A. flos-aquae*	–	Growth inhibition	Aliotta et al., 1999
5-methoxypsoralen	*Ruta graveolens*	*Synechococcus leopolensis, A. flos-aquae*	–	Growth inhibition	Aliotta et al., 1999
Alantolactone	*Inula helenium*	*O. perornata*	>100 μg mL^-1∗^	Growth inhibition	Cantrell et al., 2007
Anthraquinone	Plant extracts	*Oscillatoria perornata*	63 nM	Inhibits photosynthesis	Schrader et al., 2000, 2003
Bacillamide	*Bacillus* sp. (Jeong et al., 2003)	*M. aeruginosa, Aphanizomenon gracile, Anabaena circinalis, Anabaenopsis circularis*	29–160 μg mL^-1^	Morphological and ultrastructural changes, growth inhibition, reduction, and collapse of gas; vesicles, distortion of cell shape	Churro et al., 2009
Caffeic acid (CA)	*Hydrilla verticillata, Vallisneria spiralis*	*M. aeruginosa*	∼5 mg l^-1^	Growth inhibition	Gao et al., 2011
Chrysophanol	*Limonium myrianthum*	*O. perornata*	10 μg mL^-1∗^	Growth inhibition	Cantrell et al., 2007
*Cis*-6-octadecenoic	*Myriophyllum spicatum*	*Microcystis aeruginosa*	3.3 ± 0.4 mg l^-1^	Growth inhibition	Nakai et al., 2005
*Cis*-9-octadecenoic acids	*Myriophyllum spicatum*	*Microcystis aeruginosa*	1.6 ± 0.4 mg l^-1^	Growth inhibition	Nakai et al., 2005
Dicyclohexanyl orizane	*Oryza sativa*	*M. aeruginosa*	100 μg l^-1^ (66–80% inhibition)	Growth inhibition	Park et al., 2009
Ellagic acid	*Myriophyllum spicatum*	*M. aeruginosa*	5.1 mg l^-1^	Produced free radicals, growth inhibition	Nakai et al., 2000
Ethyl 2-methyl acetoacetate (EMA)	*Phragmites communis*	*Microcystis aeruginosa*	0.65 ± 0.13 mg l^-1^	Damage of cell membrane, ion leakage, decreased activity of antioxidants	Li and Hu, 2005
Eudesmin	*Haplophyllum sieversii*	*Oscillatoria* sp.	–	Growth inhibition	Cantrell et al., 2005
Eugeniin	*Myriophyllum bradieme*	*M. aeruginosa, Anabaena flos-aquae*	1.6 μM	Growth inhibitory activity	Saito et al., 1989
Ferulic acid (FA)	*Hydrilla verticillata, Vallisneria spiralis*	*M. aeruginosa*	∼130 mg l^-1^	Growth inhibition	Gao et al., 2011
Flindersine	*Haplophyllum sieversii*	*Oscillatoria* sp.	15.9 μM	Growth inhibition	Cantrell et al., 2005
Gallic acid	*Myriophyllum spicatum*	*M. aeruginosa*	1.0 mg l^-1^	Produced free radicals, growth inhibition	Nakai et al., 2000
Gramine	Higher plant tannin extracts (Robinson, 1967)	*M. aeruginosa*	0.5–2.1 mg l^-1^	Oxidative damage, lipid-peroxidation	Hong et al., 2009
Haplamine	*Haplophyllum sieversii*	*Oscillatoria* sp.	1.8 μM	Growth inhibition	Cantrell et al., 2005
Harmane (1-methyl–carboline)	*Pseudomonas* sp. K44-1	*Anabaena cylindrical, A. variabilis, Oscillatoria agardhii, Anacystis marina, Microcystis aeruginosa, M. viridis*	–	Cell lysis	Kodani et al., 2002
Isoalantolactone	*Inula helenium*	*O. perornata*	100 μg mL^-1∗^	Growth inhibition	Cantrell et al., 2007
L-2-azetidinecarboxylic acid (AZC)	*Polygonatum odoratum*	*M. aeruginosa, Anabaena flos-aquae*	1.6–6.3 μM (92% inhibition)	Cell growth inhibition	Kim et al., 2006
Nanaomycin A methyl ester (NAME)	*Streptomyces hebeiensis*	*M. aeruginosa*	2.97 mg l^-1^	Lytic activity, delay cell division, enlarge cell size, decreases in biomass, esterase activity, and chlorophyll-a content, lipid peroxidation, damage of cell membrane	Feng et al., 2013
Nepodin	*Limonium myrianthum*	*Oscillatoria perornata*	100 μg mL^-1∗^	Growth inhibition	Cantrell et al., 2007
Nonanoic acid	*Myriophyllum spicatum*	*Microcystis aeruginosa*	0.5 ± 0.3 mg l^-1^	Growth inhibition, loss of plasma lemma integrity	Nakai et al., 2005
Norharmane (β-carboline 9H-pyrido(3,4-b) indole)	*Synechocystis aquatilis*	*M. aeruginosa, Oscillatoria limnetica*	4.6–4.8 μg mL^-1^	Growth inhibition	Mohamed, 2013
Phenolic compounds (HHDP-di- and -tri-galloylglucose)	*Myriophyllum verticillatum*	*Anabaena variabilis*	–	Growth inhibition	Bauer et al., 2009
Physcion	*Limonium myrianthum*	*O. perornata*	>100 μg mL^-1∗^	Growth inhibition	Cantrell et al., 2007
Prodigiosin	*Serratia marcescens*	*M. aeruginosa*	1.7–8.9 μg mL^-1^	Damage of cell membranes due to strong lytic activity	Yang et al., 2013
Protocatechuic acid (PA)	*Hydrilla verticillata, Vallisneria spiralis*	*M. aeruginosa*	∼15 mg l^-1^	Growth inhibition	Gao et al., 2011
Pyrogallol	*Myriophyllum spicatum*	*M. aeruginosa*	0.65 mg l^-1^	Growth inhibition, produced radicals, oxidant damage	Nakai et al., 2000; Shao et al., 2009
Salcolin A/B	Barley straw (*Hordeum vulgare*)	*Microcystis* sp.	6.02–9.60 × 10^-5^ mol l^-1^	Intracellular ROS formation, inhibit esterase activity, leakages of cytoplasms	Xiao et al., 2014
Torachrysone	*Limonium myrianthum*	*O. perornata*	100 μg mL^-1∗^	Growth inhibition	Cantrell et al., 2007
Tryptamine	Natural/synthetic	*M. aeruginosa, A. circinalis, Anabaenopsis circularis, Leptolyngbya* sp., *Aphanizomenon gracile, Nodularia spumigena*	<4.15 μg mL^-1^	ROS production, lipid peroxidation, irreversible membrane damages	Churro et al., 2010
Vanillic acid (VA)	*Hydrilla verticillata, Vallisneria spiralis*	*M. aeruginosa*	∼60 mg l^-1^	Growth inhibition	Gao et al., 2011
β-Ionone	Algae and higher plants	*M. aeruginosa*	21.23 ± 1.87 mg l^-1^	Decrease in pigment content, thylakoids distortion, damage of PS II reaction center	Shao et al., 2011
β-sitosterol-β-D-glucoside	*Oryza sativa*	*M. aeruginosa*	100 μg l^-1^ (66–80% inhibition)	Growth inhibition	Park et al., 2009

*^∗^Lowest-complete-inhibition concentration.*

## Physical Approaches

Bloom control by physical methods generally involves mechanical removal techniques or short wavelength radiation treatment to control the incidence of cyanobacteria. The use of new and improved technologies can eliminate industrial/agricultural/ household pollutants to a certain extent to minimize the environmental pollution, including the water pollution by the incidence of harmful algal blooms. Global climate change and rising fresh water demand for multipurpose usage caused a remarkable increase in drought frequency and decreased freshwater flow rates (Paerl and Huisman, 2008; Paul, 2008). However, increasing flow rates and decreasing water residence time can remove fresh water algal blooms of a reservoir even in nutrient-rich conditions (Paerl, 2008). The artificial circulation for increased water flow is reported to suppress the blooms, but it may also cause habitat disturbance (Visser and Ibelings, 1996; Jungo et al., 2001; Huisman et al., 2004; Hudnell et al., 2010). Moreover, a solar powered circulation (SPC) has been designed to create long-distances circulation of the epilimnion (>200 m) to suppress freshwater harmful algal blooms (Hudnell et al., 2010). Data obtained from a case study of nutrient-enriched, source-water reservoirs, revealed the role of SPC in reduction of cyanobacterial peak density by about 82 and 95% during the first and second year of SPC deployment, respectively (Hudnell et al., 2010). Intensity of light and temperature play a significant role in bloom incidence as mentioned above. However, the increase in incidence of light and temperature can hardly be controlled in a large water reservoir, where as it is energy intensive in smaller water bodies. Short wavelength ultraviolet radiation can bring about a rapid degradation of the cyanotoxins MCs (Tsuji et al., 1995; Kaya and Sano, 1998). Moreover, it has been concluded that photosensitized processes may play an important role in the photochemical transformation of cyanotoxins (e.g., MC-LR; cylindrospermopsin) in the natural water (Lawton et al., 1999; Song et al., 2007; Wörmer et al., 2010; He et al., 2012). Simulated waterfalls or fountains may also be effective to control the cyanobacterial blooms in smaller water bodies; however, it requires electric-grid power constantly (Clevely and Wooster, 2007). The use of hydraulic jet cavitation may be a good approach to cyanobacterial water-bloom management (Jančula et al., 2014). Moreover, cavitation treatment can disintegrate gas vesicles of cyanobacterial cells, and can remove up to 99% cyanobacteria growing in a lake, ponds or reservoirs (Jančula et al., 2014).

## Biological Approaches

Control of cyanoblooms through biological mechanisms such as regulation of nutrient uptake or availability, alteration of normal physiology (such as a decrease in photosynthetic pigment), and/or direct feeding of cyanobacterial biomass by some aquatic organisms may be promising ways of ecological restoration (Bond and Lake, 2003; Qin et al., 2006; Zhang et al., 2008; Zhang et al., 2012, 2014). The gastropod *Radix swinhoei* can ingest cyanobacteria and survive well without loss in fecundity in the water reservoirs with cyanobacterial blooms (Zhang et al., 2012). The combined use of snails (*R. swinhoei*) and a submerged plant (*Potamogeton lucens*) in eutrophic waters can eliminate cyanobacterial bloom by minimizing the eutrophication; however, this method is under the preliminary stage due to the lack of the field study (Zhang et al., 2014). Occurrence and growth of aquatic plants are considered good candidates for limiting algal growth as the aquatic plants directly compete with algae for nutrients, light and space (Qiu et al., 2001; Wang et al., 2009). Some aquatic plants release different allelochemicals that can inhibit the growth of cyanobacteria and other phytoplanktons (Nakai et al., 2000; Körner and Nicklisch, 2002). Biodegradation using different species/strains of bacteria (**Table 3**) and other organisms may be the most efficient process to control the fate of some cyanotoxins in natural waters (Zhang et al., 2008; Manage et al., 2009; Lawton et al., 2011; Rastogi et al., 2014).

**Table 3:** Biological control of some common cyanotoxins by different bacterial isolates.

Bacterial isolates	Strains	Microcystin variants	Reference
*Arthrobacter* sp.	C6, F7, F10, R1, R4, R6, R9	LR	Manage et al., 2009; Lawton et al., 2011
*Bacillus nanhaiencis*	K-W 39	LR	Zhang et al., 2015
*Brevibacterium* sp.	F3	LR	Manage et al., 2009; Lawton et al., 2011
*Bacillus* sp.	AMRI-03, EMB	LR, RR	Alamri, 2010; Hu et al., 2012
*Bordetella* sp.	MC-LTH1	LR, RR	Yang et al., 2014a
*Methylobacillus* sp.	J10	LR, RR	Hu et al., 2009
*Microbacterium* sp.	DC8	LR	Ramani et al., 2012
*Novosphingobium* sp.	THN1	LR	Jiang et al., 2011
*Ochrobactrum* sp.	FDT5	LR	Jing et al., 2014
*Pseudomonas aeruginosa*	–	LR	Lemes et al., 2015
*Rhizobium gallicum*	DC7	LR	Ramani et al., 2012
*Rhodococcus* sp.	C1, C3	LR	Manage et al., 2009; Lawton et al., 2011
*Sphingomonas* sp.	MD-1	LR, RR, YR	Saito et al., 2003
*Sphingomonas* sp.	7CY	LR, RR, LY, LW, LF	Ishii et al., 2004
*Sphingopyxis* sp.	LH21	LR, LA	Ho et al., 2007
*Sphingopyxis* sp.	C-1	LR, RR	Okano et al., 2009
*Sphingopyxis* sp.	USTB-05	RR, YR	Wang et al., 2010; Xu et al., 2015
*Stenotrophomonas* sp.	EMS	LR, RR	Chen et al., 2010
*Stenotrophomonas acidaminiphila*	MC-LTH2	LR, RR	Yang et al., 2014b

## Research and Management

Development of wastewater research and management program is highly amenable to prevent or control the worldwide incidence of algal blooms and maintaining the ecological integrity and sustainability. Documentation of different environmental factors responsible for increased incidence of harmful cyanoblooms is crucial toward the development of demarcated management strategies. Moreover, interactive management of anthropogenic over nutrient-enrichment and global climate change is a major task for ensuring the protection and sustainability of aquatic ecosystems (Paerl et al., 2011a,b). The availability of phosphorus plays an important role in the growth of cyanobacteria and other microalgae or phytoplanktons (Schindler et al., 2008); henceforth, controlled input of phosphorus to the water reservoir may be an effective management strategy for restoration of aquatic ecosystems. In order to minimize the bloom boosting organic or inorganic nutrients coming from common practices such as excessive use of fertilizers (e.g., NPK) and detergents, prior wastewater treatment may be needed to reduce the incidence of cyanobacterial blooms (Conley et al., 2009; Paerl et al., 2011a; Jacquet et al., 2014). The modeling of different water bodies at risk of toxic blooms may be a good approach to develop a proactive algal bloom monitoring and management strategies (Tyler et al., 2009; Coad et al., 2014). Moreover, the fundamental research and quantitative ecological awareness toward the bloom incidence can be a supportive tool guiding large-scale water management against harmful bloom incidence.

## Public Awareness Approaches

Public environmental awareness (PEA) is a fundamental approach for the attainment of sustainable environment (Xu et al., 2013; Kirkpatrick et al., 2014). PEA about the incidence and harmful effects of toxic cyanoblooms may be a dynamic approach to eradicate and avoid the blooms and their toxic effects. The edifying approaches will allow people to think about their practices in their day-to-day life, such as unregulated disposal of organic/inorganic domestic wastes in the water reservoir, thereby enhancing the risks of bloom formation. As discussed elsewhere, global climate change may potentially impact the success of cyanobloom incidence. An emphasis on social practice to minimize the bloom formation and intoxication can allow intellectuals actualizing the significant development to control the environmental pollution. A change in the PEA levels in response to the increased incidence of environmental pollution is indispensable for ensuring the effective environmental protection and restoration (Xu et al., 2013). An increase in public awareness regarding the environmental sustainability and ecosystem health can inform the policy or decision makers to develop the strategies or to set-up the environmental protection laws against anthropogenic environmental pollution (such as direct disposal of domestic or industrial waste in open water reservoir such as rivers, ponds, lakes, and catchments). Moreover, various means of environmental protection program should be launched worldwide by the concerned government or non-government organization (NGO) to spread the knowledge about different environmental issues such as harmful cyanobloom incidence (Mikami et al., 1995; Palmer et al., 1998; Li et al., 2008; Várkuti et al., 2008; Xu et al., 2013).

Overall, little is known concerning the formation of cyanoblooms and production of different variants of cyanotoxin in diverse water bodies. Furthermore, each of these strategies mentioned above has their own advantages and limitations, and more extensive collaborative work is needed to control or manage the occurrence of algal blooms worldwide. Since eutrophication is considered as the most immediate environmental consequence of cyanoblooms, the uncontrolled disposal of organic/inorganic nutrients in the water reservoir through agricultural runoff or through industrial and household sewage water must be diminished or even forbidden. The establishment of several dyes or chemical based industries are the source of several blooms forming substances and therefore the government law should strictly be implemented to sanitize unwanted industrial eﬄuents before reaching the water bodies. Furthermore, the eutrophication of water reservoirs must be regularly checked for an increased prevalence of toxin producers mainly in the bloom sensitive areas of subtropical and temperate climates. Severity on global warming is also an important trigger that is likely to create toxic cyanoblooms, therefore a proper environmental management toward increasing global climate change is necessary for sustainability of the pollution-free aquatic ecosystems.

## Conclusion and Perspective

Cyanobacterial blooms are an increasing issue in both the wastewater-treatment and drinking water systems. Eutrophication and global climate change is the key factors for the occurrence of cyanoblooms all over the world. Cyanoblooms and production of several cyanotoxins in water bodies may reduce the surface/drinking water quality leading to high health risk to the organisms in aquatic ecosystems as well as wild/domestic animals and humans. A number of cyanotoxins such as MCs, nodularins, cylindrospermopsins, anatoxins, saxitoxins, and LPSs have been recognized as the major environmental contaminants in the immediate aquatic ecosystems. Control of cyanobloom using the chemical approaches can be effective; however, some algicidal/herbicides chemicals can cause secondary pollution of aquatic ecosystems. Several mitigation strategies have been tested and employed at laboratory levels; however, their efficacy to remove the blooms has not been confirmed under field environments. Establishment of effective mitigation strategies such as chemical, biological as well as public cognizance approach toward environmental awareness may be the most realistic measure to overcome the worldwide incidence of algal blooms and the attainment of a sustainable environment. Some natural algicidal compounds are really very effective to control the cyanoblooms; however, their production and availability is still very limited. The cost-effective synthesis of these biochemicals would be highly valuable to control the cyanoblooms. Furthermore, several cyanobacteria may become resistant toward certain chemicals. The use of biocides or several different biological processes against cyanoblooms may also affect other non-target aquatic organisms. Hence, common ecotoxicological impacts should be sensibly evaluated in the milieu of the lack of ecological health risk assessment. Moreover, a combined policy should strictly be regulated to diminish the bloom-boosting cause such as massive eutrophication of aquatic ecosystems by anthropogenic sources.

## Conflict of Interest Statement

The authors declare that the research was conducted in the absence of any commercial or financial relationships that could be construed as a potential conflict of interest.
